# Bovine Herpesvirus 1 U_L_49.5 Interacts with gM and VP22 To Ensure Virus Cell-to-Cell Spread and Virion Incorporation: Novel Role for VP22 in gM-Independent U_L_49.5 Virion Incorporation

**DOI:** 10.1128/JVI.00240-18

**Published:** 2018-06-13

**Authors:** Katrin Pannhorst, Huiyong Wei, Hocine Yezid, Junyun He, Shafiqul I. Chowdhury

**Affiliations:** a Department of Pathobiological Sciences, School of Veterinary Medicine, Louisiana State University, Baton Rouge, Louisiana, USA; Northwestern University

**Keywords:** U_L_49.5/gM complex, gM maturation, gM and U_L_49.5 virion incorporation, novel U_L_49.5-VP22 interaction

## Abstract

Alphaherpesvirus envelope glycoprotein N (gN) and gM form a covalently linked complex. Bovine herpesvirus type 1 (BHV-1) U_L_49.5 (a gN homolog) contains two predicted cysteine residues, C42 and C78. The C42 is highly conserved among the alphaherpesvirus gN homologs (e.g., herpes simplex virus 1 and pseudorabies virus). To identify which cysteine residue is required for the formation of the U_L_49.5/gM complex and to characterize the functional significance of the U_L_49.5/gM complex, we constructed and analyzed C42S and C78S substitution mutants in either a BHV-1 wild type (wt) or BHV-1 U_L_49.5 cytoplasmic tail-null (CT-null) virus background. The results demonstrated that BHV-1 U_L_49.5 residue C42 but not C78 was essential for the formation of the covalently linked functional U_L_49.5/gM complex, gM maturation in the Golgi compartment, and efficient cell-to-cell spread of the virus. Interestingly, the C42S and CT-null mutations separately did not affect mutant U_L_49.5 virion incorporation. However, when both of the mutations were introduced simultaneously, the U_L_49.5 C42S/CT-null protein virion incorporation was severely reduced. Incidentally, the anti-VP22 antibody coimmunoprecipitated the U_L_49.5 C42S/CT-null mutant protein at a noticeably reduced level compared to that of the individual U_L_49.5 C42S and CT-null mutant proteins. As expected, in a dual U_L_49.5 C42S/VP22Δ virus with deletion of VP22 (VP22Δ), the U_L_49.5 C42S virion incorporation was also severely reduced while in a gMΔ virus, U_L_49.5 virion incorporation was affected only slightly. Together, these results suggested that U_L_49.5 virion incorporation is mediated redundantly, by both U_L_49.5/gM functional complex and VP22, through a putative gM-independent novel U_L_49.5 and VP22 interaction.

**IMPORTANCE** Bovine herpesvirus 1 (BHV-1) envelope protein U_L_49.5 is an important virulence determinant because it downregulates major histocompatibility complex class I (MHC-I). U_L_49.5 also forms a covalently linked complex with gM. The results of this study demonstrate that U_L_49.5 regulates gM maturation and virus cell-to-cell spread since gM maturation in the Golgi compartment depends on covalently linked U_L_49.5/gM complex. The results also show that the U_L_49.5 residue cysteine 42 (C42) mediates the formation of the covalently linked U_L_49.5-gM interaction. Furthermore, a C42S mutant virus in which U_L_49.5 cannot interact with gM has defective cell-to-cell spread. Interestingly, U_L_49.5 also interacts with the tegument protein VP22 via its cytoplasmic tail (CT). The putative U_L_49.5 CT-VP22 interaction is essential for a gM-independent U_L_49.5 virion incorporation and is revealed when U_L_49.5 and gM are not linked. Therefore, U_L_49.5 virion incorporation is mediated by U_L_49.5-gM complex interaction and through a gM-independent interaction between U_L_49.5 and VP22.

## INTRODUCTION

Bovine herpesvirus type 1 (BHV-1) is an important pathogen of cattle that can cause a severe respiratory tract infection, known as infectious bovine rhinotracheitis (IBR), and abortion in pregnant cows (1, 2). In addition, BHV-1 is an important component of the bovine respiratory disease complex (BRDC), also known as shipping fever (3, 4). The BHV-1 gene product, envelope protein U_L_49.5, a glycoprotein N (gN) homolog of alphaherpesviruses, forms a disulfide-linked complex with envelope glycoprotein M (gM). Both proteins are nonessential although in the absence of either U_L_49.5 or gM, virus yield is reduced significantly (5, 6). The U_L_49.5 gene products of BHV-1, herpes simplex virus 1 (HSV-1), and equine herpesvirus 1 (EHV-1) are not glycosylated (6–8). The corresponding U_L_49.5 gene product of pseudorabies virus (PRV) is glycosylated and is termed gN (9). In PRV, gN is not essential for gM maturation in the Golgi compartment and for gM virion incorporation, but gM is necessary for gN virion incorporation (10). In contrast, formation of the U_L_49.5/gM complex is essential for BHV-1 gM maturation in the Golgi compartment (11, 12). Currently, it is not known whether BHV-1 gM and/or U_L_49.5 is necessary for each other's virion incorporation. Among the varicelloviruses, BHV-1, PRV, and EHV-1 U_L_49.5 or its gN homologs bind to the transporter associated with antigen presentation (TAP) in virus-infected cells and thereby downregulates major histocompatibility complex class I (MHC-I) cell surface expression (11, 13). However, unlike the PRV and EHV-1 proteins, BHV-1 U_L_49.5 not only binds but also degrades TAP (13).

**FIG 1:**
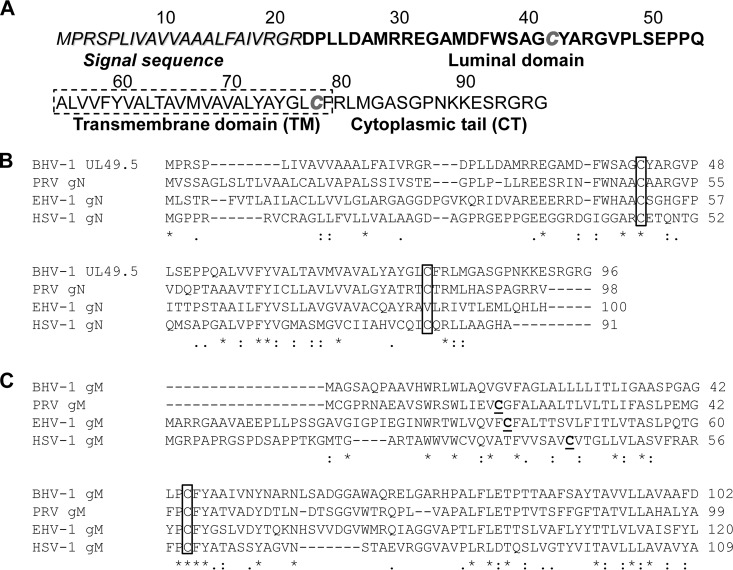
BHV-1 U_L_49.5 predicted amino acid sequence and conserved cysteine residues between the gN and gM homologs of alphaherpesviruses. (A) Predicted amino acid sequences of the BHV-1 U_L_49.5 open reading frame (ORF). Signal sequence and luminal (ecto), transmembrane, and cytoplasmic tail domains are shown; the two cysteine residues C42 and C78 are italicized. (B) Alignment of predicted amino acid sequences of BHV-1, PRV, EHV-1, and HSV-1 U_L_49.5/gN homologs. Note that C42 (boxed) is conserved in all four gN homologs and that C78 is conserved in BHV-1, PRV, and HSV-1 but not in EHV-1. (C) Alignment of predicted amino acid sequences of BHV-1, PRV, EHV-1, and HSV-1 gM homologs. Cysteines (C) that are not conserved are in bold. Conserved cysteines are boxed. Asterisks (*) indicate positions which have a single, fully conserved residue; colons (:) indicate conservation between groups of strongly similar properties; periods (.) indicate conservation between groups of weakly similar properties.

BHV-1 U_L_49.5 is a 9-kDa type I membrane protein (6). The predicted U_L_49.5 open reading frame (ORF) encodes 96 amino acids (aa) and is composed of an N-terminal signal sequence of 22 aa, an extracellular luminal domain of 32 aa, a transmembrane (TM) domain of 25 aa, and a short cytoplasmic tail (CT) of 17 aa (14) (Fig. 1A). There are two predicted cysteine residues in the BHV-1 U_L_49.5 ORF, C42 and C78. Alignment of BHV-1 U_L_49.5 amino acid sequences with the corresponding U_L_49.5 sequences of other alphaherpesviruses showed that C42, located within the luminal domain of BHV-1 U_L_49.5, is highly conserved among alphaherpesviruses (Fig. 1B). The complex between U_L_49.5 and gM is thought to be linked via disulfide bonds between cysteine residues. Since the BHV-1 U_L_49.5 C42 is highly conserved among herpesviruses (Fig. 1B), we hypothesized that the BHV-1 U_L_49.5/gM complex is mediated by the predicted U_L_49.5 residue C42 and a predicted cysteine residue in the gM ORF (Fig. 1C).

It was previously postulated that BHV-1 U_L_49.5 binds to TAP through its TM domain (11). However, it has not been possible to map the BHV-1 U_L_49.5/TAP binding domain within the TM because even a short deletion within the BHV-1 U_L_49.5 TM domain resulted in degradation of the protein (11, 15). Additionally, it was reported that in a stably transfected cell line, gM interferes with U_L_49.5-mediated TAP inhibition and MHC-I downregulation function, indicating that gM might compete with U_L_49.5 for TAP binding (12). Recently, we have reported that U_L_49.5 residues 30 to 32 (RXE motif) within the luminal domain and the U_L_49.5 CT residues together mediated maximum U_L_49.5 TAP inhibition function without affecting the covalent U_L_49.5/gM interaction (15). These findings raised the question of whether the C78 residue within the U_L_49.5 TM domain, also conserved in the PRV gN, is important for U_L_49.5-TAP interaction and thereby MHC-I downregulation.

The goal of this study was to determine whether one or both cysteine residues are required for the formation of covalently linked U_L_49.5/gM complex, gM maturation, cell-to-cell spread of the virus, and U_L_49.5 or gM virion incorporation. Additionally, we wanted to investigate whether the mutation of one or both cysteine residues affects the U_L_49.5-mediated MHC-I downregulation function.

To this end, we have constructed several BHV-1 U_L_49.5 mutants with residue C42 or C78 replaced individually or simultaneously with a serine (S) residue using a U_L_49.5 CT-null or wild-type (wt) virus as a backbone. Further, we have constructed two BHV-1 VP22 deletion mutants, one with wt U_L_49.5 and the other with a U_L_49.5 C42S mutation, and analyzed their respective levels of U_L_49.5 virion incorporation. Finally, we constructed a virus with a deletion of gM (gM-deleted) and determined its U_L_49.5 virion incorporation. The results demonstrated the following: (i) that the U_L_49.5 residue C42 but not C78 is essential for formation of the U_L_49.5/gM covalently linked complex and gM maturation in the Golgi compartment; (ii) that the U_L_49.5 C42S and U_L_49.5 CT-null mutant proteins are incorporated in the respective mutant's virion envelope but that the U_L_49.5 C42S lacking U_L_49.5 CT residues 80 to 96 is not or markedly reduced; (iii) that covalently linked U_L_49.5 and mature gM are incorporated in the virion of a VP22 deletion (VP22Δ) strain and that, however, unlinked U_L_49.5 and immature gM require VP22 for their virion incorporation; and (iv) that in the absence of U_L_49.5/gM complex, a gM-independent U_L_49.5-VP22 interaction mediated probably by U_L_49.5 CT residues 80 to 96 is essential for U_L_49.5 virion incorporation.

## RESULTS

BHV-1 U_L_49.5 forms a disulfide-linked complex with gM, which is required for gM processing in the Golgi compartment (5). BHV-1 U_L_49.5 also downregulates MHC-I cell surface expression by interacting with TAP in the endoplasmic reticulum (ER) (11). To investigate whether one or both of the cysteine residues in U_L_49.5 affects U_L_49.5-gM interaction and gM processing, the C42S and C78S mutants were generated using infectious BHV-1 wt and BHV-1 U_L_49.5 CT-null bacterial artificial chromosome (BAC) clones. In addition, a C42S/C78S/CT-null triple mutant virus with a double cysteine substitution was constructed. These mutant viruses were characterized with respect to their plaque phenotypes and growth kinetics, mutant U_L_49.5/gM complex formation, gM maturation, and U_L_49.5/gM virion envelope incorporation. In addition, effects of C42S and/or C78S mutation on the mutant U_L_49.5-mediated downregulation of MHC-I cell surface expression due to TAP inhibition were analyzed.

### Mutation of U_L_49.5 residue C42 but not C78 resulted in a growth defect and small-plaque phenotype.

**FIG 2:**
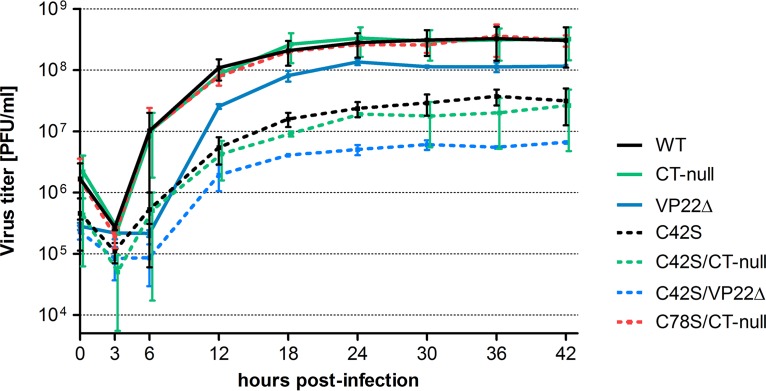
Growth kinetics of BHV-1 U_L_49.5 mutants in MDBK cells. One-step growth kinetics of BHV-1 wild-type (wt), CT-null, VP22Δ, C42S, C42S/CT-null, C42S/VP22Δ, and C78S/CT-null viruses. Each data point represents the average of duplicate samples obtained from two separate infections. Error bars represent standard errors of the means.

**FIG 3:**
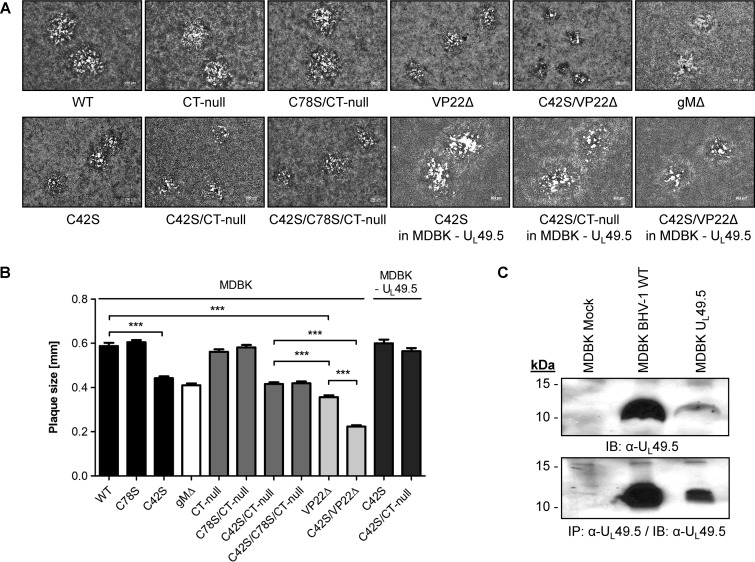
Plaque morphology of BHV-1 U_L_49.5 mutants in MDBK cells and MDBK cells expressing wt U_L_49.5. (A) Representative images of plaque morphology of BHV-1 wt, U_L_49.5 CT-null, C42S, C78S/CT-null, C42S/CT-null, C42S/C78S/CT-null, VP22Δ, C42S/VP22Δ, and gMΔ viruses in MDBK cells. For comparison, plaque morphologies of C42S, double mutant C42S/CT-null, and double mutant C42S/VP22Δ viruses produced in the wt U_L_49.5-expressing MDBK cell line (U_L_49.5-MDBK) are shown. Plaque sizes were measured at 48 hpi. (B) Bar graph showing comparative plaque sizes produced by BHV-1 wt, U_L_49.5 C78S, C42S, gMΔ, CT-null, C78S/CT-null, C42S/CT-null, C42S/C78S/CT-null, VP22Δ, and C42S/VP22Δ viruses. For comparison, plaque sizes produced by U_L_49.5 C42S and double mutant C42S/CT-null viruses in a wt U_L_49.5-expressing MDBK cell line (U_L_49.5-MDBK) are shown. Error bars represent standard errors of the means. ***, *P* < 0.001. (C) Analysis of U_L_49.5 expression in a stable MDBK U_L_49.5-expressing cell line compared with the level in wt virus-infected MDBK cells, as determined by immunoblotting (IB) or by immunoprecipitation (IP) with anti-U_L_49.5 antibody.

To examine whether the C42S or C78S substitution within U_L_49.5 affected viral replication kinetics and virus yield in infected MDBK cells, one-step growth curves of C42S, C78S/CT-null, C42S/CT-null, VP22Δ, C42S/VP22Δ, wt, and CT-null viruses were determined. The results showed that viral growth kinetics of CT-null and C78S/CT-null are almost identical to wt kinetics (Fig. 2). However, both the C42S and double C42S/CT-null mutant viruses replicated with a 10-fold-reduced virus yield compared to that of their parental wt and CT-null viruses (Fig. 2). As shown in Fig. 3A and B, average plaque sizes produced in MDBK cells by C42S and double C42S/CT-null mutant viruses were significantly smaller than those of their respective parental wt and CT-null viruses. However, C78S (Fig. 3B) and C78S/CT-null mutant viruses had approximately the same diameters as the parental wt and CT-null viruses (Fig. 3A and B). The double cysteine C42S/C78S mutant virus produced plaques very similar to those produced by C42S and double C42S/CT-null mutant viruses, suggesting that the C42S mutation affected plaque size (Fig. 3A and B). In the wt U_L_49.5-expressing, stable MDBK cell line (MDBK-U_L_49.5), the C42S and C42S/CT-null viruses produced plaques of wild-type size (Fig. 3A and B), and they replicated with a 5-fold-higher titer than in noncomplementing MDBK cells (data not shown). However, the virus yield was still 5-fold lower than that of the wild-type virus, which could be due to a low level of U_L_49.5 expression by the stable cell line (Fig. 3C). Therefore, these results indicated that the growth defects (smaller plaque phenotype and 10-fold lower yield) of the C42S, C42S/CT-null, and C42S/C78S mutant viruses were due to the replacement of the U_L_49.5 C42 residue with a serine residue and not due to another mutation elsewhere in the genome.

### U_L_49.5 residue C42 but not C78 is required for the formation of covalently linked U_L_49.5/gM complex and gM maturation in the Golgi compartment.

**FIG 4:**
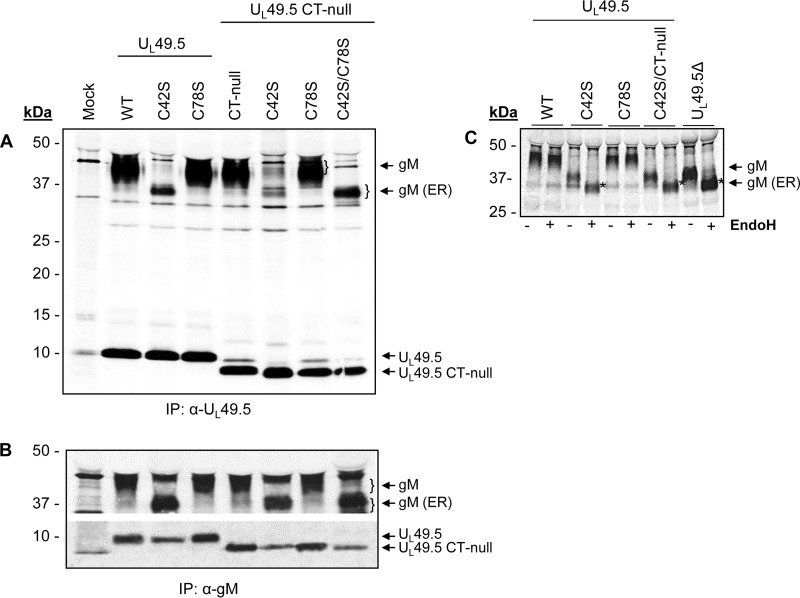
Analysis of U_L_49.5-gM interaction by radioimmunoprecipitation assay. ^35^S-labeled lysates from mock-infected or BHV-1 U_L_49.5 mutant virus-infected MDBK cells were immunoprecipitated with anti-U_L_49.5-specific (A) or anti-gM-specific (B) polyclonal antibodies, separated by SDS-PAGE, and visualized by autoradiography. Note that there is a nonspecific 43-kDa faint band in the mock-infected sample in both panels A and B; this band is also present in the wt- and mutant virus-infected lysate samples but is visible only when the gM (43 kDa) is not processed (C42S mutants). Also, in panel A anti-U_L_49.5 antibody precipitated a nonspecific 9-kDa faint band in the mock-infected sample, and this band is also visible in the CT-null lysates. (C) ^35^S-labeled lysates from various mutant virus-infected MDBK cells were immunoprecipitated with anti-gM-specific antibody and digested with EndoH (+). The untreated samples (−) were included as controls. EndoH-sensitive, immature gM is marked by asterisks.

To determine whether U_L_49.5 residues C42, C78, or both are essential for covalently linked U_L_49.5-gM interactions and gM processing in the Golgi compartment, ^35^S-labeled C42S, C78S, C42S/CT-null, C78S/CT-null, and C42S/C78S/CT-null mutant proteins expressed in the respective mutant virus-infected cells were immunoprecipitated with anti-U_L_49.5 and anti-gM antibodies and analyzed by Western blotting. As controls, wt and CT-null virus-infected cell lysates were similarly analyzed. As shown in Fig. 4A, U_L_49.5-specific antibody immunoprecipitated 9-kDa U_L_49.5 wt, C42S, and C78S proteins, but 8-kDa U_L_49.5 CT-null, C42S/CT-null, C78S/CT-null, and C42S/C78S/CT-null proteins were immunoprecipitated from the corresponding wt and mutant viruses. In addition, the antibody coimmunoprecipitated 43-kDa mature gM-specific proteins from wt, CT-null, C78S, and C78S/CT-null virus-infected cell lysates. However, the U_L_49.5-specific antibody coimmunoprecipitated 36-kDa immature gM-specific proteins from the C42S, C42S/CT-null, and C42S/C78S/CT-null mutant virus-infected cell lysates unlike results with the wt and C78S mutant (Fig. 4A). Notably, a vastly reduced level of the 36-kDa immature gM was coimmunoprecipitated by the U_L_49.5-specific antibody. As expected, gM-specific antibody immunoprecipitated the 43-kDa mature gM from wt, CT-null, C78S, and C78S/CT-null virus-infected cell lysates. Similar to results with immunoprecipitation with the anti-U_L_49.5 antibody, a 36-kDa gM protein was also immunoprecipitated from the C42S, C42S/CT-null, and C42S/C78S/CT-null virus-infected cell lysates (Fig. 4B). In addition, the anti-gM-specific antibody coimmunoprecipitated the corresponding U_L_49.5-specific 9-kDa C42S and C78S proteins and the 8-kDa CT-null, C42S/CT-null, C78S/CT-null, and C42S/C78S/CT-null proteins. However, the levels of U_L_49.5 C42S, C42S/CT-null, and C42S/C78S/CT-null proteins coimmunoprecipitated with the anti-gM antibody were reduced compared with the levels of the wt, CT-null, and C78S/CT-null proteins (Fig. 4B).

We hypothesized that the 43-kDa proteins detected in the wt, C78S, and CT-null virus-infected lysates are the mature Golgi-processed gM proteins and that the 36-kDa band detected in the C42S virus-infected lysate is the immature gM. Therefore, we determined their endoglycosidase H (EndoH) sensitivity. As expected, results showed that the 43-kDa mature gM protein (Golgi apparatus-processed) was resistant to EndoH digestion (Fig. 4C), but the 36-kDa immature gM protein was EndoH sensitive.

**FIG 5:**
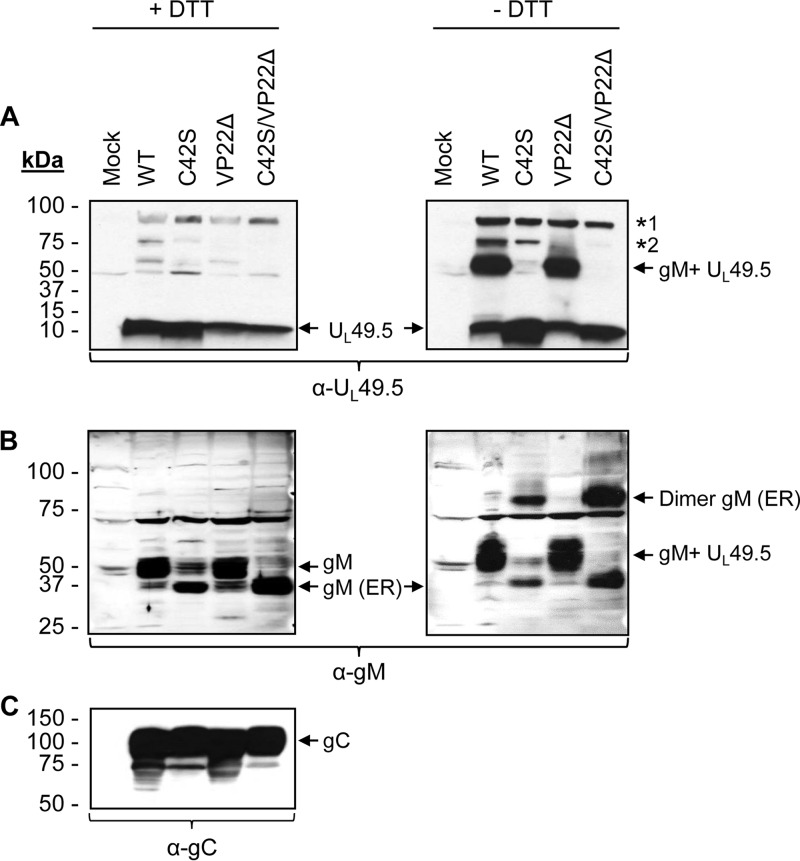
Analysis of BHV-1 U_L_49.5/gM covalently linked complex and gM maturation. Lysates of BHV-1 wt, C42S, VP22Δ, and the double mutant U_L_49.5 C42S/VP22Δ virus-infected MDBK cells were separated by SDS-PAGE under reducing (+DTT) or nonreducing (−DTT) conditions and immunoblotted with either anti-U_L_49.5-specific (A) or anti-gM-specific (B) antibodies. Note that under nonreducing conditions (−DTT), the U_L_49.5 C42S mutant protein expressed by the C42S and double U_L_49.5 C42S/VP22Δ mutant viruses did not comigrate with gM (A), indicating the loss of the C42-mediated covalent bond between U_L_49.5 C42S and gM. An approximately 90-kDa band (*1) was detected in all of the virus-infected cell lysates. In addition, an approximately 80-kDa band (*2) was detected in wt- and U_L_49.5 C42S virus-infected lysates. Note that in the case of the C42S and double U_L_49.5 C42S/VP22Δ mutant viruses, only the 36-kDa immature form of gM (gM-ER) and its 72-kDa homodimer were detected (B). (C) Immunoblotting of the mock-infected and corresponding virus-infected lysates with anti-gC-specific antibody served as a loading control.

To confirm that the U_L_49.5 C42S mutation disrupts the formation of the U_L_49.5/gM covalently linked complex, wt, C42S, VP22Δ, and C42S/VP22Δ virus-infected cell lysates were subjected to SDS-PAGE under both reducing and nonreducing conditions and analyzed by immunoblotting with either anti-U_L_49.5 or anti-gM antibodies. The results shown in Fig. 5 demonstrated that under reducing conditions (with dithiothreitol [+DTT]), wt U_L_49.5 migrated as a 9-kDa band (Fig. 5A), while under nonreducing conditions (−DTT), a large portion of the wt U_L_49.5 comigrated with gM as a 52-kDa heterodimer (Fig. 5A). However, under both reducing and nonreducing conditions, the mutant U_L_49.5 C42S protein migrated by itself as a 9-kDa band (Fig. 5A).

Interestingly, under both reducing and nonreducing conditions, anti-U_L_49.5-specific antibody recognized two higher-molecular-mass proteins of approximately 92 kDa in all virus-infected cell lysates and an approximately 80-kDa protein in wt- and C42S-infected lysates. The 80-kDa band was not detected in VP22Δ and U_L_49.5 C42S/VP22Δ virus-infected cell lysates. These bands were more prominent under the nonreducing conditions. Both the 92-kDa and 80-kDa proteins were absent in the mock-infected cell lysate (Fig. 5A). When the identical blot was immunoblotted with the gM-specific antibody, both proteins were absent (Fig. 5B). Thus, the 92-kDa band might represent a heterodimeric complex of the approximately 9-kDa U_L_49.5 protein plus the approximately 82-kDa TAP1 protein (UniProt accession number A6QPZ6). Currently, the identity of the 80-kDa protein is not known.

Further, as shown in Fig. 5B under nonreducing conditions, the anti-gM antibody recognized an additional approximately 72-kDa protein in the C42S and C42S/VP22Δ virus-infected cell lysates but not in the mock-, wt-, and VP22Δ-infected cell lysates. It is highly likely that the 72-kDa protein represents a covalently linked homodimer of the 36-kDa immature gM.

To validate further that the U_L_49.5 C42S mutation alone can be attributed to the disruption of the U_L_49.5/gM covalent interaction required for gM maturation, we analyzed gM maturation in the U_L_49.5 C42S mutant-infected MDBK-U_L_49.5-expressing cell line. As expected, the effect of the U_L_49.5 C42S mutation on gM maturation was complemented to its mature 43-kDa molecular mass by the U_L_49.5-expressing cell line. However, the rescue or complementation of the 43-kDa protein was at a reduced level (data not shown). As noted above with respect to U_L_49.5 C42S mutant virus yield, the complementation at a reduced level is due to a lower level of U_L_49.5 expression by the stable cell line, probably due to a lower copy number of the expressed U_L_49.5 gene than during wt virus infection. Therefore, these results indicated that the C42S mutation alone is responsible for the defective gM maturation and growth defect (small-plaque phenotype and 10-fold-lower virus titer) of the mutant U_L_49.5 C42S virus.

Taken together, the results indicated (i) that U_L_49.5 residue C42 but not C78 is required for the formation of a covalent U_L_49.5-gM complex, (ii) that gM was processed to the mature 43-kDa protein only when it was covalently linked to wt U_L_49.5, and (iii) that in the absence of covalently linked U_L_49.5/gM complex, the immature gM can form a covalently linked homodimer.

### U_L_49.5 luminal domain residue C42 and CT residues 80 to 96 are redundantly essential for U_L_49.5 virion incorporation.

**FIG 6:**
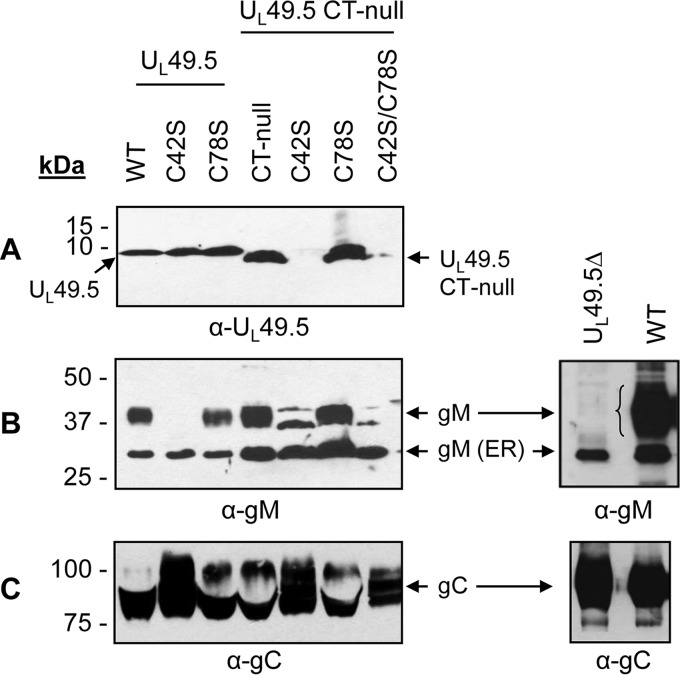
Analysis of U_L_49.5 mutant viruses for U_L_49.5 and gM virion incorporation. Virions were partially purified through a 30% sucrose cushion and ultracentrifugation as described previously (31). Virion lysates of BHV-1 wt and various U_L_49.5 cysteine residue mutants were separated by a 5 to 20% gradient SDS-PAGE gel and immunoblotted with anti-U_L_49.5-specific (A) or anti-gM-specific (B) antibodies. Immunoblotting with anti-gC-specific antibody (C) served as a loading control. The 43-kDa mature and the 36-kDa immature gM proteins are shown. Note that in the case of U_L_49.5 C42S/CT-null virus, an approximately 43-kDa protein was detected by the gM-specific antibody; similar bands were not EndoH resistant (data not shown).

To determine the effects of U_L_49.5 residue C42 and C78 substitutions with or without the additional U_L_49.5 CT-null mutation on mutant U_L_49.5 and gM virion incorporation, purified wt and CT-null, C42S, C78S, C42S/CT-null, C78S/CT-null, C42S/C78S/CT-null, and U_L_49.5Δ mutant virions were analyzed by immunoblotting with either U_L_49.5- or gM-specific antibodies (Fig. 6). The results showed that even though similar levels of U_L_49.5 and mutant U_L_49.5 proteins were present in each of the mutant virus-infected cell lysates (Fig. 4A), the double C42S/CT-null and triple C42S/C78S/CT-null mutant U_L_49.5 proteins were not incorporated or were markedly reduced in their respective virion particles. However, the wt U_L_49.5, CT-null, C42S, C78S, and C78S/CT-null mutant proteins were incorporated into their respective virion particles (Fig. 6A). Therefore, only the simultaneous C42S and CT-null mutations but not the individual C42S and CT-null mutations affected U_L_49.5 virion incorporation (Fig. 6A). These results indicated that both U_L_49.5 residue C42 and U_L_49.5 CT residues 80 to 96 are essential for U_L_49.5 virion incorporation.

### Both C42S and U_L_49.5Δ mutant viruses incorporated immature gM in the virion envelope.

The results presented in Fig. 6B showed that in the case of wt, CT-null, C78S, and double C78S/CT-null mutant viruses, both mature and immature gM proteins were incorporated into the virion envelope. The immature gM was incorporated into the envelope of C42S, double C42S/CT-null, triple C42S/C78S/CT-null, and U_L_49.5Δ viruses (Fig. 6B). Therefore, incorporation of the immature gM in the virion envelope appears to be independent of its covalently linked interaction with U_L_49.5.

### The U_L_49.5 CT residues 80 to 96 most likely interact with VP22.

**FIG 7:**
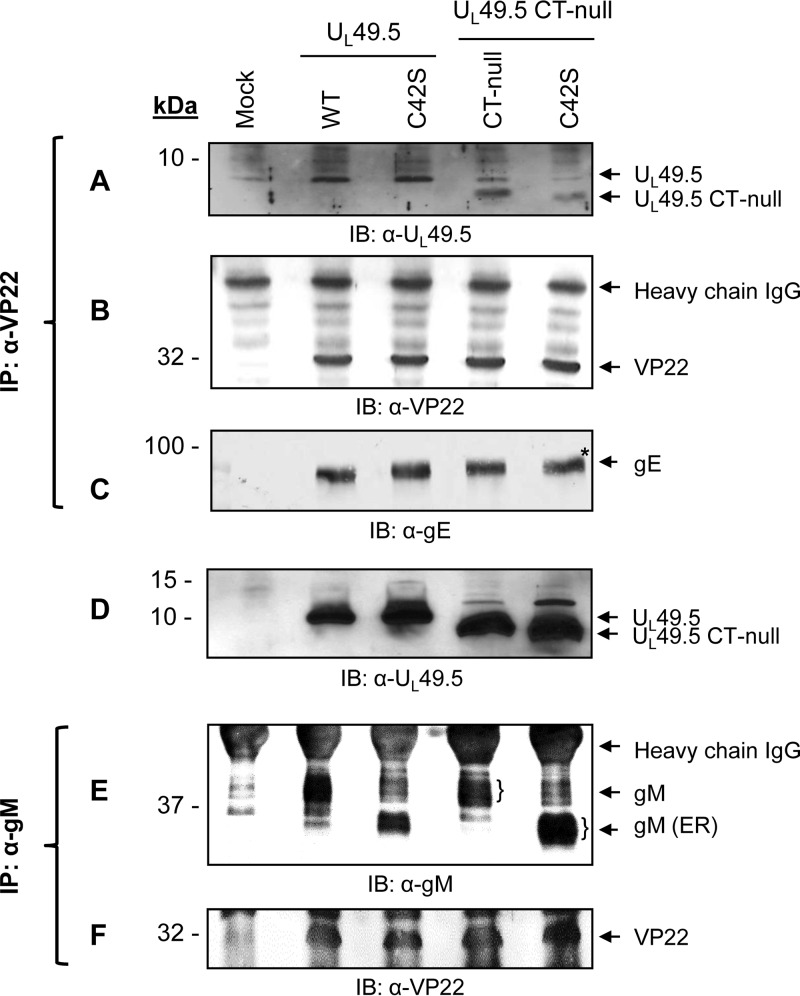
Analysis of U_L_49.5-VP22 and gM-VP22 interactions by coimmunoprecipitation. Infected cell lysates of mock, BHV-1 wt, C42S, CT-null, and C42S/CT-null viruses were immunoprecipitated with anti-VP22 antibody. Immunoprecipitated proteins were separated by SDS-PAGE, and Western blotting of membranes from the identical gels was performed with anti-U_L_49.5 (A), anti-VP22 (B), and anti-gE (C) antibodies. The levels of VP22 and gE immunoprecipitated and/or coimmunoprecipitated, respectively, with the anti-VP22 antibody from wt and mutant U_L_49.5 virus-infected cell lysates and visualized by anti-VP22 and anti-gE antibodies served as loading controls. (D) As a cell lysate control, an immunoblot developed with anti-U_L_49.5-specific antibody of mock-, BHV-1 wt-, and mutant U_L_49.5-infected cell lysates is shown. (E and F) Infected cell lysates of mock, BHV-1 wt, C42S, CT-null, and C42S/CT-null viruses were immunoprecipitated with anti-gM antibody. Western blotting of the immunoprecipitated proteins was performed with anti-gM-specific and anti-VP22-specific antibodies.

VP22 in HSV-1 and PRV is well known for its interaction with a number of envelope proteins (gM, gE, and gD) (16) and tegument protein VP16 (17, 18). We hypothesized that U_L_49.5 CT might be interacting with VP22 and thus play a role in the U_L_49.5 C42S virion incorporation. Therefore, we determined whether the mutant U_L_49.5 proteins expressed by C42S, CT-null, and double C42S/CT-null mutant viruses are coimmunoprecipitated with anti-VP22 antibody (Fig. 7). Since VP22 also interacts with gE, which was not manipulated in the U_L_49.5 mutants, we compared the levels of U_L_49.5, gE, and VP22 that are coimmunoprecipitated by anti-VP22 antibody from the corresponding virus-infected cell lysates. The results showed that in both wt and U_L_49.5 mutant virus-infected lysates, VP22-specific antibody immunoprecipitated or coimmunoprecipitated similar levels of VP22 and gE (Fig. 7B and C). However, the anti-VP22 antibody coimmunoprecipitated a reduced level of dual U_L_49.5 C42S/CT-null mutant protein compared with the corresponding level of C42S and CT-null mutant proteins (Fig. 7A). Since VP22 also interacts with gM in alphaherpesviruses (16, 17), we determined additionally the level of VP22 coimmunoprecipitated with anti-gM antibody in the wt, U_L_49.5 C42S, CT-null, and C42S/CT-null mutant virus-infected cell lysates with the anti-gM antibody. As shown in Fig. 7, regardless of gM maturation status (Fig. 7E), the levels of VP22 coimmunoprecipitated by the anti-gM antibody from the corresponding virus-infected cell lysates were very similar (Fig. 7F). Taken together, these data indicated (i) that U_L_49.5 CT residues 80 to 96 most likely interact with VP22, which is revealed only in the absence of covalent U_L_49.5/gM complex, and (ii) that neither C42S nor double C42S/CT-null mutations affected the gM-VP22 interaction.

### In the absence of covalent U_L_49.5/gM complex, incorporation of U_L_49.5 in the virion envelope is probably mediated by interaction of U_L_49.5 CT residues 80 to 96 and VP22.

**FIG 8:**
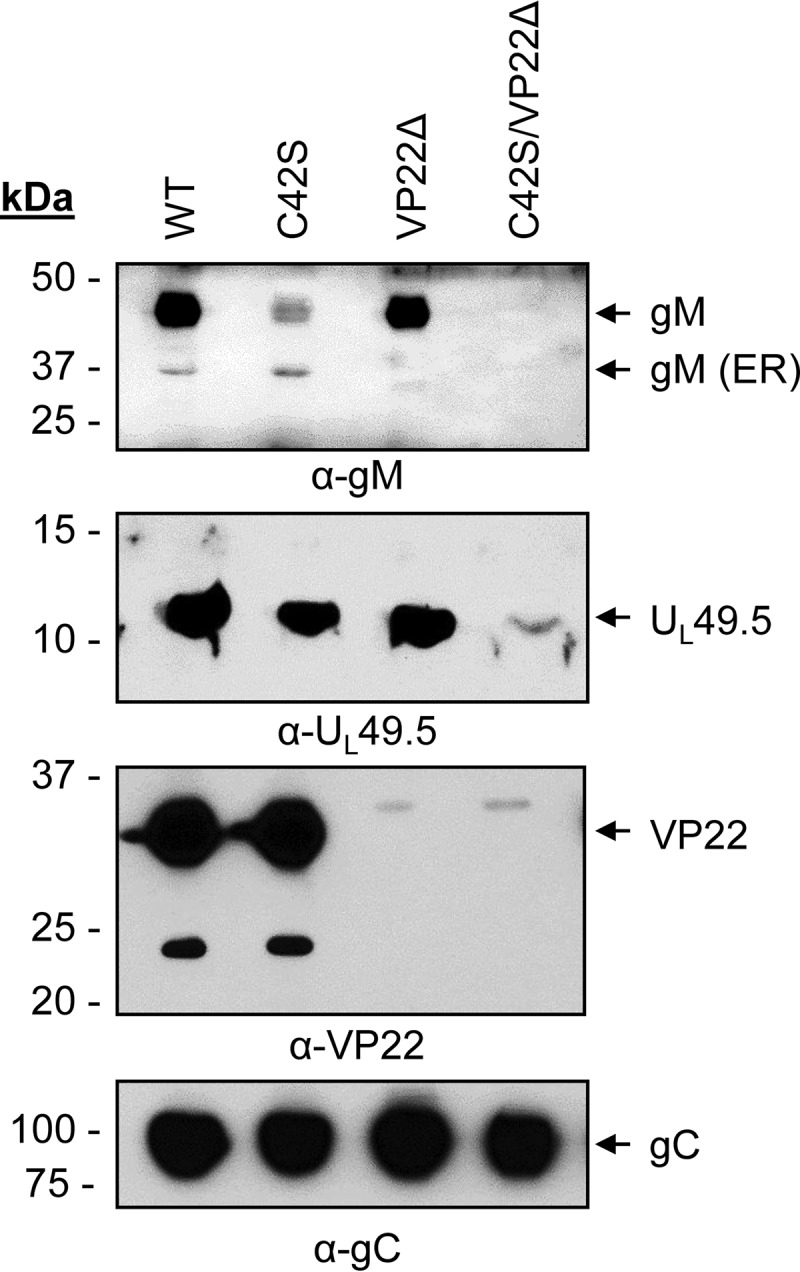
Analysis of U_L_49.5 and gM incorporation in the virion envelope of C42S, VP22Δ, and U_L_49.5 C42S/VP22Δ mutant viruses compared with levels in the wt. Partially purified virions were separated by SDS-PAGE and immunoblotted with anti-gM-, anti-U_L_49.5-, anti-VP22-, or anti-gC-specific antibodies. Amounts of proteins loaded for wt, C42S, VP22Δ, and C42S/VP22Δ are 29.4, 31.2, 29.4, and 29.4 μg, respectively.

**FIG 9:**
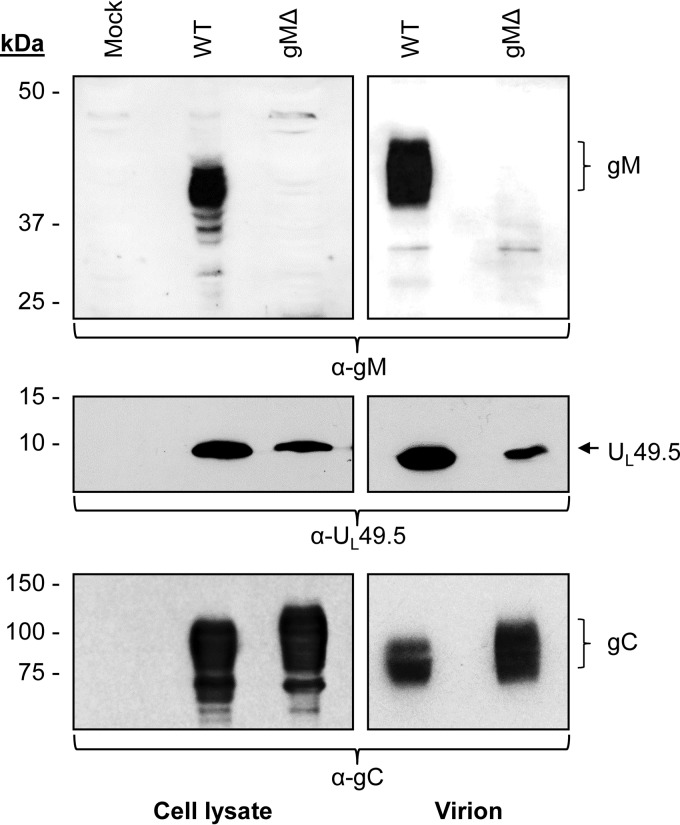
Analysis of U_L_49.5 incorporation in the virion envelope of a gMΔ mutant virus. Partially purified virions and infected cell lysates of BHV-1 wt and gMΔ viruses were separated by SDS-PAGE and immunoblotted with anti-gM- or anti-U_L_49.5-specific antibodies. Immunoblotting with anti-gC-specific antibody served as loading control.

To determine whether VP22 interaction with U_L_49.5 CT residues 80 to 96 plays an essential role in mutant U_L_49.5 C42S protein virion incorporation, a double C42S/VP22Δ and VP22Δ mutant virus were constructed. We predicted (i) that mutant U_L_49.5 C42S protein virion incorporation in the presence of U_L_49.5 CT residues 80 to 96 and VP22 will not be affected, and (ii) that the C42S mutant protein containing U_L_49.5 CT residues 80 to 96 but expressed by a VP22Δ mutant virus would be defective. As predicted, a significantly reduced level of U_L_49.5 C42S, expressed in the backbone of a VP22Δ mutant virus, was incorporated into the virion envelope (Fig. 8). However, the results also showed that the 36-kDa immature gM expressed by the C42S mutant virus was incorporated into the virion, but the immature gM in context of the U_L_49.5 C42S/VP22Δ virus was not. This raised the alternative possibility that lack of VP22-immature gM interactions led to the defective U_L_49.5 C42S incorporation into the virion. To exclude this possibility, we constructed a gM-deleted virus and determined whether U_L_49.5 expressed in the absence of gM is incorporated in the virion envelope. As shown in Fig. 9, U_L_49.5 expressed in the backbone of a gM-deleted virus was incorporated in the virion though at a slightly reduced level.

### Neither the individual U_L_49.5 cysteine residue mutations nor the combined mutations had an effect on MHC-I cell surface expression in mutant virus-infected cells.

**FIG 10:**
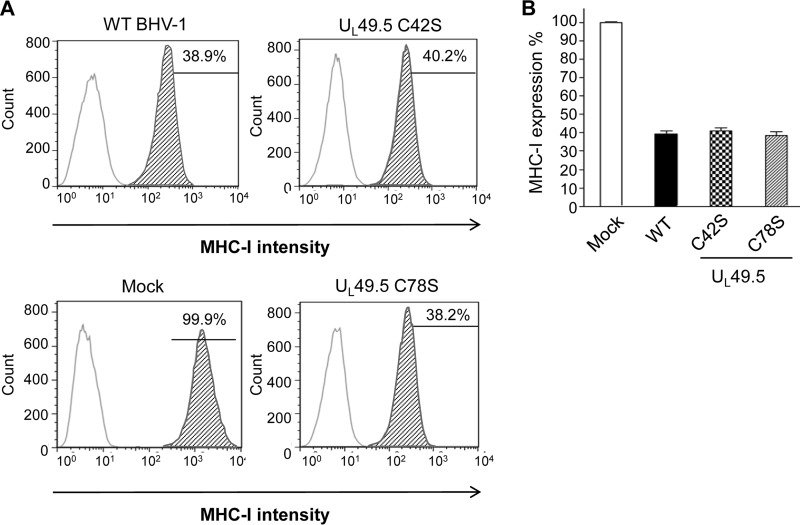
FACS analysis of MHC-I cell surface expression in wt and U_L_49.5 mutant virus-infected MDBK cells. Cells were infected with wt, C42S, and U_L_49.5 C78S mutant viruses. MHC-I cell surface expression was detected at 18 hpi with a monoclonal anti-MHC-I antibody in combination with an anti-mouse FITC antibody and determined by FACS analysis. (A) Representative graphs of MHC-I cell surface expression of virus-infected cells. (B) Mean of three independent experiments of MHC-I expression in virus-infected cells.

To determine the effects of U_L_49.5 residue C42 and C78 substitutions on U_L_49.5-mediated TAP inhibition or MHC-I downregulation, we compared MHC-I cell surface expression in the C42S, C78S, and double C42S/C78S mutant virus-infected cells with that of wt virus-infected cells. Fluorescence-activated cell sorting (FACS) analysis results clearly showed that the C42S and C78S mutations, either individually or combined, did not abrogate U_L_49.5-mediated MHC-I downregulation (Fig. 10).

## DISCUSSION

**FIG 11:**
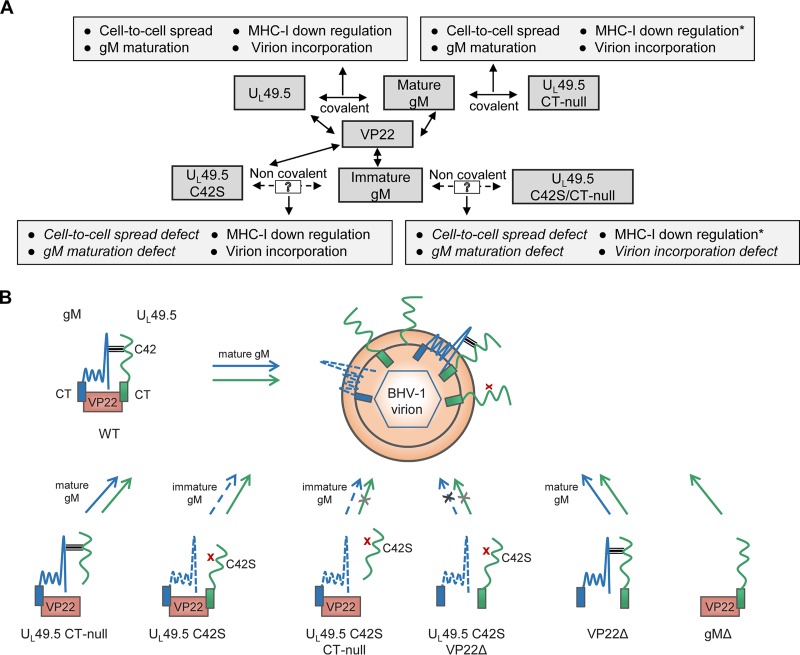
(A) Proposed scheme showing U_L_49.5 C42 and CT residue interactions with gM and VP22, respectively, and their functional implications. Double-headed solid arrows indicate the interaction between the two proteins, and single-headed solid arrows link the specific protein or its interactions to known functions. The dashed arrow indicates a putative noncovalent mutant U_L_49.5/gM interaction. A 13% increase in MHC-I cell surface expression due to U_L_49.5 CT-null mutation (15) is also indicated (*). (B) Schematic summary of U_L_49.5 and gM virion incorporation of different U_L_49.5, gM, and VP22 mutant viruses. Note that covalently linked U_L_49.5/gM complex is essential for gM maturation and cell-to-cell spread of virus but not for U_L_49.5-mediated MHC-I downregulation. Note that in the absence of covalently linked U_L_49.5 and gM complex (U_L_49.5 C42S mutation), virion incorporation of U_L_49.5 C42S and immature gM proteins is not affected. However, simultaneous U_L_49.5 C42S/CT-null mutation or U_L_49.5 C42S mutation in the backbone of VP22Δ virus affected the mutant U_L_49.5 C42S and immature gM incorporation in the virion. This indicates that in the absence of covalently linked U_L_49.5/gM complex, U_L_49.5 CT-VP22 interaction was required for U_L_49.5 C42S virion incorporation. As predicted, in the gM-deleted virus, U_L_49.5 was incorporated in the virion because in the absence of gM, U_L_49.5 CT interaction with VP22 promoted U_L_49.5 virion incorporation. The horizontal gray bars indicate the U_L_49.5-gM interaction, a red cross indicates the disruption of the U_L_49.5-gM interaction due to the C42S mutation, a black cross on the arrow indicates no virion incorporation, and a light gray cross on the arrow indicates either no or markedly reduced virion incorporation.

We conducted these studies to determine the following: (i) which of the two U_L_49.5 cysteine residues (C42 and C78) is required for formation of the covalently linked U_L_49.5/gM complex and gM maturation; (ii) whether one or both of the proteins play a role in each other's virion incorporation; and (iii) how U_L_49.5 residue C42S and C78S mutations affect MHC-I downregulation. The results of this study determined (i) that the covalently linked U_L_49.5/gM complex is necessary for BHV-1 gM processing in the Golgi compartment; (ii) that the U_L_49.5 residue C42S substitution mutation and not the C78S mutation affected the formation of the covalently linked U_L_49.5/gM complex; and (iii) that the covalently linked U_L_49.5/gM complex is also necessary for efficient cell-to-cell spread of the virus and efficient virus replication. Notably, C42S mutant virus produced 30% smaller plaques and replicated with more than 10-fold-reduced virus yield. The results also showed the following: (iv) that in the absence of the covalently linked U_L_49.5/gM complex, both immature gM and U_L_49.5 are incorporated into the virion envelope in a VP22-dependent manner; (v) that while the individual C42S or CT-null mutations had no effect on U_L_49.5 virion incorporation, dual C42S and CT-null mutations affected the U_L_49.5 C42S/CT-null virion incorporation; and (vi) that in both U_L_49.5 C42S and C78S mutant virus-infected cells, MHC-I cell surface expression was not affected. For a better understanding of the results and the discussion below, the interactions of wt and mutant U_L_49.5 with gM and VP22 and their effects on the U_L_49.5/gM complex and/or their virion incorporation are shown in Fig. 11.

Previously, by using stable cell lines expressing gM or both U_L_49.5 and gM, Lipinska et al. (12) reported that the U_L_49.5/gM complex formation was required for gM maturation in the Golgi compartment. They also reported that in cells infected with a virus with a deletion of the U_L_49.5 TM domain (BHV-1 U_L_49.5ΔTM), gM was not processed in the Golgi compartment. Since the U_L_49.5 TM domain contains one of the two cysteine residues of U_L_49.5 (C78) and since U_L_49.5/gM complex formation involves covalently linked disulfide bonds, they suggested that the U_L_49.5 C78 residue is essential for U_L_49.5/gM complex formation and gM processing. However, in that study, the status of the mutant U_L_49.5 protein expressed by U_L_49.5Δ TM virus was not analyzed. Recently, we reported that deletion of the U_L_49.5 TM domain resulted in degradation of the mutant U_L_49.5 protein and that gM expressed by the mutant U_L_49.5Δ TM virus was not processed (15). Here, we have characterized the U_L_49.5 C42S and C78S mutant viruses for U_L_49.5/gM complex formation and gM processing. Our results demonstrate that the U_L_49.5 residue C42 and not C78 was required for the formation of the covalently linked U_L_49.5/gM complex and that in the absence of the covalently linked U_L_49.5/gM complex, the C42S mutant virus produced smaller plaques and replicated with reduced virus yield. Nevertheless, in agreement with Lipinska et al. (12), we found that gM processing in the Golgi compartment is dependent on the covalently linked U_L_49.5/gM complex formation. Therefore, in BHV-1, U_L_49.5 (gN homolog) is a dominant determinant of gM maturation in the Golgi compartment. However, the opposite is true for HSV and PRV because gM is required for transport and/or processing of gN in the Golgi compartment (10, 19, 20).

Our results also indicate the following in BHV-1: (i) that U_L_49.5 and gM incorporation into the virion may occur without a covalently linked U_L_49.5/gM complex and that the uncomplexed U_L_49.5 and gM virion incorporation require VP22; (ii) that in the absence of covalently linked U_L_49.5/gM complex, U_L_49.5 CT residues 80 to 96 are essential.

In alphaherpesviruses, the tegument protein VP22 is known to interact with multiple viral proteins and thereby regulate their cellular translocations (17, 18). In HSV-1 (17) and PRV (16, 18), VP22 binds to both gE and gM and bridges a complex between gE and gM. Hence, we predicted that VP22 also interacts (i) with U_L_49.5 in a gM-independent manner and (ii) with the immature gM. Therefore, VP22 may play a redundant role in U_L_49.5 and gM virion incorporation. Additionally, we hypothesized that U_L_49.5 CT residues most likely interact with VP22, and this interaction may be essential for gM-independent U_L_49.5 virion incorporation. We proved these possibilities in five different ways: (i) by showing that the levels of U_L_49.5 C42S/CT-null coimmunoprecipitated by an anti-VP22 antibody from the mutant virus-infected cell lysates is reduced; (ii) by showing that the level of U_L_49.5 C42S virion incorporation in a double U_L_49.5 C42S/VP22Δ mutant virus is vastly reduced; (iii) by showing that in the absence of gM (gMΔ virus) U_L_49.5 is incorporated in the virion; (iv) by showing that the immature gM expressed by the C42S and C42S/CT-null mutant viruses was incorporated into the virion but that the immature gM in the backbone of the C42S/VP22Δ virus was not; and (v) by determining that both the mature and immature gM proteins interacted with VP22 with similar efficiencies.

In summary, these results revealed a redundant role of VP22 in the virion incorporation of wt U_L_49.5/gM complex and a novel but essential role for both U_L_49.5 C42S and immature gM virion incorporation when they are not covalently linked.

Even though the covalently linked U_L_49.5/gM complex was not essential for U_L_49.5 or gM virion incorporation, the complex was essential for cell-to-cell spread of virus. Recently, El Kasimi and Lippe reported that U_L_49.5 regulates gM translocation and cell-to-cell fusion at the basolateral cell surface (19). In human herpesvirus 6 (HHV-6), gN was required for gM maturation, and the gN-gM complex interacted with v-SNARE protein vesicle-associated membrane protein 3 (VAMP3) in infected cells (21), which is known to facilitate membrane fusion (22). It is noteworthy that while U_L_49.5 downregulates MHC-I cell surface expression during BHV-1 infection to evade cellular immune responses, it may also regulate the post-Golgi transport of gM and/or the U_L_49.5/gM complex to promote viral cell-to-cell spread and to avoid the circulating neutralizing antibodies. Therefore, in light of the above reports of gN/gM complex in HSV-1 and HHV-6, it could be interesting to determine whether the BHV-1 U_L_49.5/gM complex also interacts with v-SNARE, with or without VP22. In conclusion, our U_L_49.5 mutational study determined that the U_L_49.5/gM functional complex was necessary for efficient cell-to-cell spread of the virus but not for U_L_49.5 and gM virion incorporation and MHC-I downregulation. Importantly, the U_L_49.5 mutational study revealed a previously unidentified gM-independent novel, functional interaction of VP22 with U_L_49.5.

## MATERIALS AND METHODS

### Cells and wt U_L_49.5-expressing cell line.

The MDBK cell line was maintained in Dulbecco's modified Eagle's medium (DMEM) supplemented with 5 to 10% heat-inactivated fetal bovine serum (FBS). The MDBK cell line expressing wt U_L_49.5 was generated as described previously (15) and maintained in DMEM supplemented with FBS as above, but supplemented additionally with blasticidin as described earlier (15).

### Virus and bacterial strains.

The BHV-1 Cooper (Colorado-1) strain was obtained from the American Type Culture Collection (ATCC VR-864) and low-passage-number viral stocks were maintained. Reconstituted BHV-1 Cooper BAC-excised virus and BHV-1 U_L_ 49.5 CT-null BAC-excised virus were generated previously (15, 23). BHV-1 virus with a deletion of U_L_49.5 was a kind gift from E. J. Wiertz (Leiden University, The Netherlands). Infectious BHV-1 wt and BHV-1 U_L_49.5 CT-null BAC clones were maintained in Escherichia coli strain DH10B. E. coli strain SW105 (kindly provided by N. G. Copeland) was used for Red recombination.

### Antibodies.

Horseradish peroxidase (HRP)-conjugated donkey anti-rabbit IgG (Thermo), mouse anti-BHV-1 gC monoclonal antibody (MAb) F2 (24), HRP-conjugated goat anti-mouse IgG (Invitrogen), mouse anti-MHC-I Ab (H58A; Veterinary Medical Research and Development [VMRD]), and fluorescein isothiocyanate (FITC)-conjugated rat anti-mouse IgG (ebioscience) were purchased from commercial sources.

The anti-BHV-1 gM-specific, anti-BHV-1 U_L_49.5-specific, and anti-BHV-1 gEectodomain-specific rabbit polyclonal antibodies were produced previously (15, 25). Rabbit and goat anti-VP22 antibodies were produced commercially (Cocalico Biologicals) by using a cocktail of two peptides corresponding to predicted VP22 aa 22 to 34 ([H]-RENSLYDYESGSD-[OH]) and aa 244 to 258 ([H]-TSGGESRLRGERARP-[OH]) conjugated to polyethylene glycol.

Rabbit and goat anti-BHV-1 gE cytoplasmic tail-specific polyclonal antibody was generated (Cocalico Biologicals) using purified E. coli-expressed BHV-1 gE aa 451 to 564 as described earlier (26).

### Construction of mutant viruses.

Table 1 includes a list of viruses used in this study.

**TABLE 1:** Viruses used and or/constructed in this study

Virus	Description
Wild type	BHV-1
C42S	U_L_49.5 residue C42 replaced with a serine
C78S	U_L_49.5 residue C78 replaced with a serine
C42S/C78S	Dual U_L_49.5 C42S and C78S mutations
CT-null^*a*^	U_L_49.5 cytoplasmic tail residues truncated/deleted
C42S CT-null	Dual U_L_49.5 C42S and CT-null mutations
C78S CT-null	Dual U_L_49.5 C78S and CT-null mutations
C42S/C78S CT-null	Triple U_L_49.5 C42S, C78S, and CT-null mutations
U_L_49.5Δ^*b*^	BHV-1 with U_L_49.5 (gN homolog) deletion
VP22Δ	BHV-1 with tegument protein VP22 (U_L_49 gene) deletion
U_L_49.5 C42S/VP22Δ	U_L_49.5 C42S mutation in a VP22Δ virus
gMΔ	BHV-1 with glycoprotein M (gM) deletion

a Reference 15.

b Kind gift of E. J. Wiertz (Leiden University, The Netherlands).

### BAC mutagenesis to generate U_L_49.5 C42S, C78S, double C42S/C78S, double C42S/CT-null, double C78S/CT-null, and triple C42S/C78S/CT-null mutant viruses.

**FIG 12:**
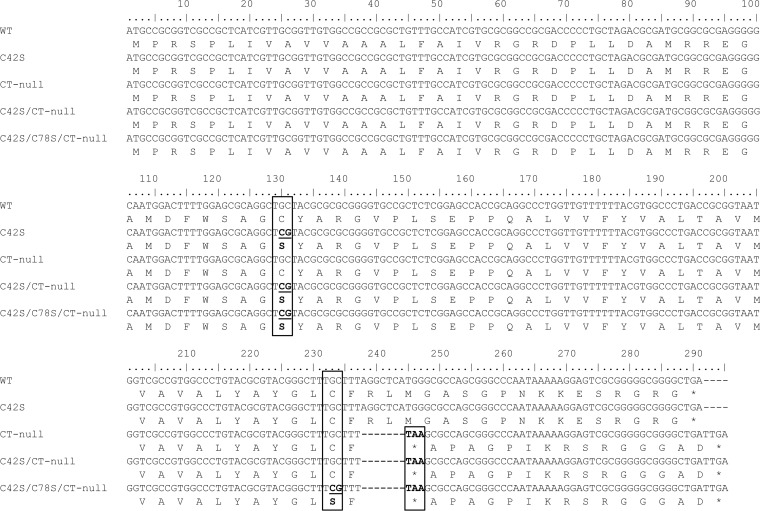
Alignment of the U_L_49.5 nucleotide sequences determined from the designated mutants. Sequences of the U_L_49.5 mutants were compared with the U_L_49.5 sequence of BHV-1 wt (GenBank accession number JX898220). Boxes at nucleotide (nt) positions 129 to 131 and 232 to 234 indicate the codon for the amino acid cysteine (C) at positions 42 and 78, respectively, that are mutated to serine (S; boldface). The dashed lines and box at U_L_ nt positions 238 to 244 and 245 to 247 indicate the deletion of two codons followed by the incorporation of a stop codon (TAA; boldface), respectively.

As shown in Table 2, primer pairs specific for serine substitutions at BHV-1 U_L_49.5 residues C42 (C42S), C78 (C78S), and both C42 and C78 (C42S/C78S) were synthesized (IDT). The first and second steps of Red-mediated mutagenesis were performed using SW105 competent cells harboring pBHV-1 BAC (wt) or pBHV-1 BAC U_L_49.5 CT-null infectious clones as described earlier (23). Reconstituted BAC-containing or BAC-excised mutant viruses were then generated as previously described (23). BAC-excised U_L_49.5 mutant viruses designated C42S, C78S, double C42S/CT-null, double C78S/CT-null, and triple C42S/C78S/CT-null mutant viruses (Table 1) were plaque purified and verified further by sequencing (Fig. 12) and immunoblotting.

**TABLE 2:** PCR primers used for generation of BHV-1 U_L_49.5 cysteine residue mutants and for colony identification

Primer function and name	Sequence (5′ to 3′)^*a*^
Mutagenesis	
C42S F	5′-**GATGCGGCGCGAGGGGGCAA***TGGACTTTTGGAGCGCAGGCtcgTACGCGCGCGGGGTGCCGCT*AGGATGACGACGATAAGTAGGG-3′
C42S R	5′-**GGGCCTGCGGTGGCTCCGAG***AGCGGCACCCCGCGCGCGTAcgaGCCTGCGCTCCAAAAGTCCA*CAACCAATTAACCAATTCTGATTAG-3′
C78S F	5′-**CGCGGTAATGGTCGCCGTGG***CCCTGTACGCGTACGGGCTTtcgTTTAGGCTCATGGGCGCCAG*AGGATGACGACGATAAGTAGGG-3′
C78S R	5′-**ACTCCTTTTTATTGGGCCCG***CTGGCGCCCATGAGCCTAAAcgaAAGCCCGTACGCGTACAGGG*CAACCAATTAACCAATTCTGATTAG-3′
C78S CT- null F	5′-**CGCGGTAATGGTCGCCGTGG***CCCTGTACGCGTACGGGCTTtcgTTTTAAGCGCCAGCGGGCCC*AGGATGACGACGATAAGTAGGG-3′
C78S CT-null R	5′-**CCCCGCGACTCCTTTTTATT***GGGCCCGCTGGCGCTTAAAAcgaAAGCCCGTACGCGTACAGGG*CAACCAATTAACCAATTCTGATTAG-3′
Verification^*b*^	
U_L_49.5 F	5′**-**AGAGCGCCAGCGAGTCGGGCTC-3′
U_L_49.5 R	5′**-**AACCGGGCCATGGCAAGCGAGTC-3′

a BHV-1 U_L_49.5-specific sequences are shown in bold uppercase letters; the italicized sequences of forward (F) and reverse (R) primers, respectively, are complementary to each other in inverse orientation. Lowercase nucleotides code for serine instead of cysteine. Underlining indicates the pEPkan-S-specific sequences.

b Primers used to PCR amplify the U_L_49.5 ORF and its downstream sequences for identification of BHV-1 U_L_49.5 BAC mutants from the selected kanamycin-sensitive colonies for sequencing.

### Construction of VP22-deleted BHV-1 mutants.

**FIG 13:**
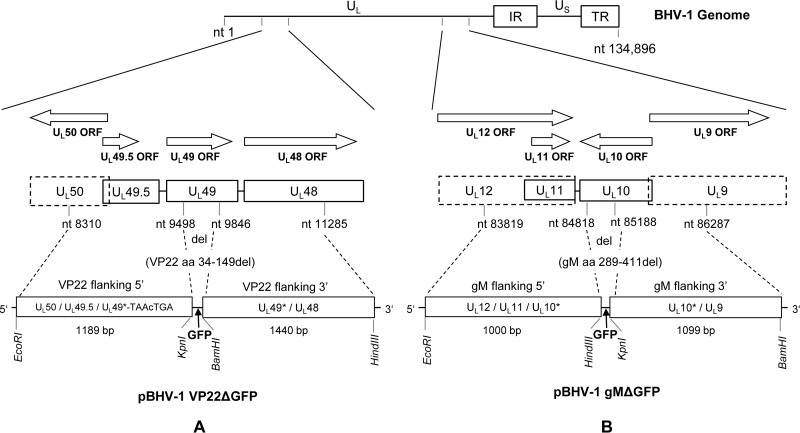
BHV-1 genomic structure and schematic map of VP22 and gM deletion plasmids (pBHV-1 VP22Δ GFP and pBHV-1 gMΔ GFP). The genomic organization of BHV-1 depicted at the top consists of unique long (U_L_) and unique short (U_S_) regions and two repeat regions (internal repeat [I_R_] and terminal repeat [T_R_]). (A) Localizations of the U_L_49 gene (VP22) and its flanking U_L_50, U_L_49.5, and U_L_48 genes are shown. In plasmid pBHV-1 VP22Δ GFP, coding sequences for amino acids 34 to 149 of VP22 are deleted (nt 9499 to 9845) and two stop codons (uppercase residues in TAAcTGA) and KpnI and BamHI restriction sites are incorporated immediately downstream of the deletion site for the insertion of the eGFP gene cassette. (B) Localizations of the U_L_10 gene (gM) and its flanking U_L_12, U_L_11, and U_L_9 genes are shown. In plasmid pBHV-1 gMΔ GFP, nt 84819 to 85187 coding for gM amino acid residues 289 to 411 were deleted, and HindIII and KpnI restriction sites are incorporated at the deletion locus for the insertion of the eGFP gene cassette.

BHV-1 VP22 is encoded by the UL49 gene (GenBank accession number JX898220). To generate a VP22-deleted BHV-1 mutant (BHV-1 VP22Δ), a chimeric 2,666-bp-long DNA fragment was first synthesized and cloned into the EcoRI (5′)/HindIII (3′) sites of pUC57, resulting in a VP22 deletion plasmid (pBHV-1 VP22Δ) (Genscript USA, Inc., NJ, USA). The 2,666-bp EcoRI/HindIII fragment consists of the following (5′ to 3′): an EcoRI site at the 5′ end, followed by 1,189-bp nucleotide sequence comprising a partial U_L_50 ORF, the full U_L_49.5 ORF, and partial U_L_49 ORF sequence (nucleotides [nt] 8310 to 9498; GenBank accession JX898220). This sequence was followed by a 25-bp non-BHV-1 sequence consisting of two stop codons (in two different reading frames; in boldface in **TAA**C**TGA**) and the KpnI/BamHI restriction sites, fused to a 1,440-bp nucleotide sequence comprising the partial carboxy-terminal U_L_49 ORF and partial U_L_48 gene sequences (nt 9846 to 11285), followed by the restriction site HindIII at the 3′ end (Fig. 13A and Table 3). In the resulting plasmid, pBHV-1 VP22Δ, nt 9499 to 9845 of the U_L_49 gene, which code for aa 34 to 149 of the VP22 protein, were deleted, and two stop codons were inserted immediately after the VP22 residue 33 (Fig. 13A and Table 3). The deletion of VP22 residues 34 to 149 and the insertion of the stop codons were verified by sequencing. Finally, an approximately 2-kb KpnI/BamHI fragment containing an enhanced green fluorescent protein (eGFP) expression cassette (26) was inserted in the corresponding KpnI/BamHI sites of pBHV-1 VP22Δ, resulting in pBHV-1 VP22Δ GFP. BHV-1 VP22Δ virus was generated by cotransfection/homologous recombination of pBHV-1 VP22Δ GFP with full-length BHV-1 wt virus DNA. A recombinant, plaque-purified BHV-1 VP22Δ virus, verified by sequencing and immunoblotting analyses, was selected for further study.

**TABLE 3:** Plasmid design for homologous recombination

Plasmid	Upstream flanking sequence			Spacer	Downstream flanking sequence		
	5′ Restriction site (sequence and name)	BHV-1 sequence (position)^*a*^	3′ Restriction site (sequence and name)		5′ Restriction site (sequence and name)	BHV-1 sequence (position)^*a*^	3′ Restriction site (sequence and name)
pBHV-1 VP22Δ	GAATTC (EcoRI)	CAAGACAAA…TCCGGCTCG (8310–9498)	**TAA**C**TGA**GGTACC (Stop-Stop-KpnI)^*b*^	CCGCGC	GGATCC (BamHI)	GTTCAGCGC…GTTTTCGGG (9846–11285)	AAGCTT (HindIII)
pBHV-1 gMΔ	GAATTC (EcoRI)	CGGTCCGTG…GTCTCCTTA (83819–84818)	AAGCTT (HindIII)	CCGCGC	GGTACC (KpnI)	TGCCACCAG…CGCGTGACC (85188–86287)	GGATCC (BamHI)

a Nucleotide (nt) positions refer to GenBank accession number JX898220; VP22 open reading frame, nt 9400 to 10176; gM open reading frame, nt 84816 to 86051.

b Stop codons are in boldface.

To generate a BHV-1 recombinant virus with dual U_L_49.5 C42S and VP22 deletions (U_L_49.5 C42S/VP22Δ), a U_L_49.5 C42S/VP22Δ vector was constructed. Briefly, the primer pairs U_L_49.5 C42S-VP22Δ forward (F) and U_L_49.5 C42S-VP22Δ reverse (R) (Table 4) and full-length genomic DNA of U_L_49.5 C42S virus DNA constructed above (template) were used to amplify the 1,191-bp fragment with EcoRI and KpnI sites at the 5′ and 3′ ends, respectively (Table 4). The 1,191-bp EcoRI/KpnI fragment was then cloned into the corresponding EcoRI/KpnI sites of the pBHV-1 VP22Δ GFP plasmid construct (Fig. 13A) described above. In the resulting plasmid clone, pU_L_49.5 C42S/VP22Δ GFP, the UL49.5 C42S mutation (TGC →TCG) was incorporated, and the nucleotide sequences coding for VP22 aa 34 to 149 were deleted. The C42S mutation in the plasmid pU_L_49.5 C42S VP22Δ GFP was verified by amplifying the entire U_L_49.5 ORF by PCR using U_L_49.5 upstream and downstream sequence-specific forward and reverse primers (Table 4) and sequencing. Subsequently, a U_L_49.5 C42S/VP22Δ GFP virus was generated by homologous recombination of pU_L_49.5 C42S/VP22Δ GFP DNA with full-length BHV-1 wt virus DNA. A recombinant double U_L_49.5 C42S/VP22Δ GFP mutant virus was plaque purified two times and verified further by sequencing and immunoblotting.

**TABLE 4:** PCR primers for generation of BHV-1 U_L_49.5 C42S/VP22Δ mutant

Primer function and name (restriction site)	Sequence^*a*^	Position (nt)^*b*^
Cloning		
VP22/34-149 del F (EcoRI)	5′-GC*GAATTC***CGCAAGACAAAGCGGCAGGGCTCC**-3′	8308–8331
VP22/34-149 del R (KpnI)	5′-GCGCTG*GGTACC*TCAGTTA**CGAGCCGGACTCGTAGTCATAGAGGCTG**-3′	9471–9498
Verification^*c*^		
U_L_49.5 F	5′-AGCGAGTCGGGCTCACAGCAGC-3′	8917–8938
U_L_49.5 R	5′-AACCGGGCCATGGCAAGCGAGTC-3′	9388–9410

a BHV-1-specific sequences are shown in boldface letters, italicized sequences are integrated restriction sites, and underlined sequences are integrated stop codons.

b Positions are based on GenBank accession number JX898220.

c Primers used for verification of the recombinant BHV-1 by sequencing.

### Construction of a gM-deleted BHV-1 mutant.

The U_L_10 gene encoding the envelope glycoprotein gM is transcribed from the complementary strand of the BHV-1 genome and is flanked on the left by U_L_11 and U_L_12 (3′ end) and on the right (5′ end) by U_L_9 (Fig. 13B). To generate a gM-deleted BHV-1 mutant (BHV-1 gMΔ), a gM deletion vector (pBHV-1 gMΔ) was generated. Briefly, a chimeric 2,100-bp-long DNA fragment was synthesized (Genscript) to include the following (5′ to 3′): an EcoRI site followed by a 1,000-bp sequence comprising a partial U_L_12 ORF, the full U_L_11 ORF, the authentic stop codon of the gM ORF (nt 83819 to 84818; GenBank accession number JX898220,), a HindIII site, six additional nucleotides (CCGCGC), and a KpnI site followed by a 1,099-bp sequence comprising partial U_L_10 and partial U_L_9 ORFs (nt 85188 to 86287) and a BamHI restriction site. This 2,100-bp EcoRI-BamHI fragment was cloned into the corresponding EcoRI/BamHI sites of pUC57 vector (GenScript). In the resulting plasmid, nt 84819 to 85187 coding for gM residues 289 to 411 were deleted and replaced with HindIII/KpnI sites, which allowed insertion of an approximately 2-kb HindIII/KpnI fragment containing an eGFP expression cassette (27), resulting in plasmid pBHV-1gMΔ eGFP (Fig. 13B). BHV-1 gMΔ virus was generated by homologous recombination of pBHV-1 gMΔ eGFP with full-length BHV-1 wt virus DNA. A recombinant BHV-1 gMΔ virus, verified by sequencing and immunoblotting analyses, was selected for further study.

### Viral growth kinetics and plaque size determination.

One-step growth curve assays were performed twice as described earlier (27). Briefly, for each virus and time point (see below), 20 T25 flasks containing 4 × 10^6^ MDBK cells/flask were seeded. The prechilled (4°C) cells were infected with various viruses at a multiplicity of infection (MOI) of 5 and adsorbed for 1 h at 4°C. Following adsorption and washing, 4 ml of medium was added to each flask, and one flask was frozen immediately for each virus sample (0 h) at −80°C. The remaining flasks were incubated further at 37°C in a CO_2_ incubator, and samples were frozen as described above at 3, 6, 12, 18, 24, 30, 36, and 42 h postinfection (hpi). Virus titers at these time points were determined by standard plaque assay as described earlier (28).

To determine the average plaque size of each mutant, two wells of a six-well plate containing confluent monolayers of MDBK cells or MDBK cells expressing wt U_L_49.5 were infected with 80 to 100 PFU of mutant viruses and overlaid with 1.6% carboxymethyl cellulose (CMC) at 2 hpi. At 48 hpi, the cells were fixed (10% formaldehyde) and stained with crystal violet. Average plaque size of wt and mutant viruses was calculated by measuring approximately 50 randomly selected plaques of each virus under a microscope with a graduated ocular objective, as described previously (15).

### Radiolabeling of mock- or virus-infected MDBK cell proteins, SDS-PAGE, and immunoprecipitation/immunoblotting analysis.

The method for [^35^S]methionine-cysteine labeling of mock- or virus-infected MDBK cells and immunoprecipitation of virus-specific proteins using protein A-Sepharose/virus protein-specific antibody was described previously (29). For the analysis of gM, virus-infected cell lysates and immunoprecipitates were incubated at 60°C in reducing sample buffer containing 100 mM dithiothreitol (DTT) as described previously (15) and separated by sodium dodecyl sulfate-polyacrylamide gel electrophoresis (SDS-PAGE). Unless otherwise mentioned in the figure legend, the SDS-PAGE was performed under reducing conditions. For SDS-PAGE under nonreducing conditions, sample buffer without DTT was used. For all other samples, cell lysates were prepared as described previously (29), and immunoprecipitates were boiled for 5 min in reducing sample buffer containing β-mercaptoethanol and separated by SDS-PAGE. Immunoprecipitated/SDS-PAGE-separated proteins were visualized by autoradiography or by immunoblotting as described earlier (15).

### EndoH digestion.

Endoglycosidase H (EndoH) digestion was performed as described previously (30). The digested samples were subjected to SDS-PAGE, and labeled proteins were visualized by autoradiography.

### FACS analysis of MHC-I cell surface expression.

MDBK cells either mock infected or infected with BHV-1 wt, U_L_49.5 C42S, or C78S mutant virus were collected at 18 hpi, blocked with IgG-free bovine serum albumin (BSA), incubated with mouse anti-bovine MHC-I antibody (Ab), and subsequently stained with FITC-conjugated rat anti-mouse Ab and analyzed by flow cytometry as described previously (15). MDBK cells infected with the respective viruses were stained by FITC-conjugated mouse IgG2a and used as isotype controls.

### Statistical analysis.

Normality of distribution of the examined variables was evaluated by a D'Agostino-Pearson omnibus normality test. Statistical significance of plaque size variations between the mutant and wt viruses was determined using a one-way analysis of variance (ANOVA) followed by Tukey's multiple-comparison test using Graph Pad Prism (GraphPad Software, La Jolla, CA, USA). A *P* value of ≤0.05 was considered statistically significant.
